# A Foreword, with Some Hospital Reminiscences

**Published:** 1917-04-21

**Authors:** 


### A FOREWORD,

WITH

### SOME HOSPITAL REMINISCENCES.

(Abstract of an Address by Sir Henry Burdett to the Bedfordshire Hospital Guild at Bedford, April 13, 1917.)

As I sat listening to the lady speakers I became impressed with a fact that was driven home to me with telling force. On active duty I went over to the United States in the autumn, and came back assured that women are setting men an example in the States and here at home, which I hope the men will follow. There is no doubt that women on the whole do more personal service in proportion to their number than the men do. Why should the women, who are now to have the vote, and are going to rule, or regulate, or reform us, and govern us, why should they have this monopoly in the exercise of the right of everyone of us in the days of health to render thanks to Almighty God for these good gifts by personal service on behalf of the sick? I see that you are mostly women: there are some men present, and therefore I should like to urge this point. Cannot each of you see that the exercise of this right must make all the difference when illness comes to you, bringing, it may be, much pain or peril to your life. If you have been a member of the Guild in the days of health, and by your work have recognised your thankfulness and indebtedness to the Great Giver of all good, and made it your privilege and pleasure to give personal service every year, to help to alleviate the suffering and provide means for the treatment of your neighbours in the hospital? I may tell you as an example for the whole country that H.M. the King is an absolute believer in personal service in the days of health. He carries out his belief in practice, deliberately paying visits to hospitals, and spending hours with the sick every year, and the Queen has been associated with him in this good work. As a matter of fact, there are few laymen who know as much about hospitals or are better able to give a competent opinion as to the quality and conduct of the management and administration of our hospitals than our present King. If you once begin personal service in such a cause you will never give it up willingly afterwards.

To-day it is a great privilege and pleasure to come to Bedfordshire, because this county in a way is one which has a will of its own. (Laughter.) Some people are pleased to say you are obstinate, and if you do not do what some people wish, they go on to say you are stupid, but experience teaches that when one is called stupid and obstinate we may be getting much nearer to the truth and closer to business than by the ordinary way. I hope you will stick to your independence. Now that we are fighting this war for freedom, surely the people in this country should be free, and if the people in this county recognise that they must be self-governed, and will have self-government, they will have pleasant places to live in. Because then, after the war, the people will not permit the continuance of bad dwellings, insanitation and many preventable ills which our poorer neighbours ought not to but do suffer from at present. I have suggested only this week in print—so I need not repeat it here—that those of us who are non-fighters, *i.e.*, the people in the Homeland, must awake and become alive to things. What are we going to do when our warriors come back? If they come back and find our Churches and ourselves asleep, without activity, exhibiting no demonstrable proof of our faith by the quickening activity of Church services everywhere, the recognition that the Spirit of God is in His Temple, and that all who are responsible for the services in the churches and chapels realise that they are His ministers, must not our neglect and supineness cause a set-back to the awakening of our soldiers and warriors to their spiritual nature? The message that I have to deliver to-day, as a special message, is that everybody amongst us has a great responsibility to discharge, before the great election which will come after the war, when we shall have to think out, and be prepared to know, how we are going to vote, and what we are going to vote for. We must suffer no more the continuance of those preventable ills—including under-payment for labour, and all the social troubles which our poorer neighbours have had to undergo in the past—or else we cannot be faithful to the principles which we profess, which have made us suffer the sacrifice of our nearest and dearest with the conviction that what we are fighting for is right. So much so is that the case that I, for one, feel that it is not for us to-day to pray to Almighty God that He will give us victory, for the war is being fought by the Allies, as we hope, to uphold the teaching of Christ, and so we can pray with all our hearts for our men out there, and ask Him that He will lead them to victory as His soldiers and servants. If we in the Homeland and our warriors are His soldiers and servants, and those are the principles for which we are fighting, we cannot remain asleep, but must wake up feeling that individually we have our responsibility and must prepare to secure that we Britons must have a completely clean slate so far as the avoidable ills existing to-day are concerned immediately after the war.

I would like to add that I have been led into this digression because of Bedfordshire and its position. I have always known Bedfordshire as the one county which went its own way, and I hope you will continue to do so. If I wanted evidence that that is good, even from my own point of view, the fact that I am here to-day is due to the circumstance that you obstinately persisted and decided, when you wanted to have a League of Mercy, to have one of your own rather than that form of personal service for the sick of which I am the parent. As organiser of that League for King Edward, I may say it was never intended, and it is not the policy of the League of Mercy to come down into the

**SOME HOSPITAL REMINISCENCES**—(*continued*).

counties and ask them to collect money, giving 1 per cent. to the County hospital and the rest to the London hospitals. If you have your organisation, it is for you to decide what you will do with your own money. If you think you would like to contribute something to London hospitals in accordance with your knowledge of how many of your people go there in difficult cases, that is a matter for you, the Bedfordshire branch, who collect the money, to decide. I think that should be made perfectly clear. It is a great thing to have this Guild here. Another reason why I at once accepted the invitation to come was that I regard these Guilds as an essential part of the ideal of the modern hospital. They are a vitalising part, because they give to a great number of people, rendering personal service, an inside view of the hospital and its work. The more this policy is pursued, the more certain is it that the spirit of the hospital will be in touch with the feelings and requirements and wishes of the patients and the community, so that the voluntary hospital itself must immeasurably benefit as well as the patients.

The modern hospital in its essence is a small republic. It has to be self-contained and has to be run upon definite lines. In fact, in the case of the larger hospitals especially, the responsibility of the heads of the chief departments is so great that they are apt to be unaware that they are really confined within four walls, although they keep their institution efficient, and, it may be, progressively efficient, but sometimes they do not get all the good that they might get if they had a wider vision, and made it their business to know and understand and follow all the developments in the outside hospital world. That is one great difficulty felt nowadays when making the appointment to an important matronship.

I recall the position of affairs in the early days when I began my hospital work in 1868, and I shall have been working continuously for hospitals during fifty years if I live until August. That work has been to me a great privilege and an enormous personal benefit. I should think there is nothing so good as hospital experience to widen our views, to increase our sympathies, and to fit us for work in the world. The modern hospital is an evolution, a progressive development through science, through social workers, and through the war. If we take the war first, has it not been indeed a developer of hospitals and treatment? Is it not a marvellous fact to those of us who look to the financial side of the hospital and believe in the voluntary system, that despite all the claims of the war, the innumerable demands, and the actual gifts of money which every one of us has to face everywhere—is it not cheering and wonderful how glad people seem to be to give to hospitals still? We have people coming forward—a continuous flow of people, and especially women—who want to help with the sick and the wounded by making all kinds of things for use in hospitals. And the more the demand, the more has been the supply and the readiness of workers to help. I can assure you that to some of us inside who have to think out and see how more hospital beds can be provided, where they can be placed, where the money is to be obtained, and how we can be ready to receive an increasing number of our warriors when they come back from the Front wounded, now that our forces are really getting to close quarters, and are going to have progressive fighting, it is marvellous that, no matter what the demand is, it is supplied with a rapidity for which we can thank God and take courage.

Take the hygienic, administrative, and scientific sides of the development of hospitals. I will show you what our hospitals were once by going back to 1868, and briefly describing the state of affairs then. It is quite extraordinary how great and numerous have been the developments. An ordinary provincial hospital, the Queen's Hospital, Birmingham, where I first held the post of superintendent, was attached to a medical school and had a medical staff of ten members; to-day there are thirty-two. In 1868 there were no trained nurses, and as far as night nurses were concerned, we had to get non-resident women, who accepted 10s. a week to sleep on a chair in the hospital wards instead of sleeping in their own beds at home. At that time there was the idea among some physicians as famous as Dr. Fleming, the discoverer of aconite, that "night nurses" were a doubtful advantage. What do you want them for? The night is made for sleep. Who wants a lot of women moving about, disturbing the patients? Let the patients alone, and give Nature a chance. That was one view of night nurses then.

We had mostly middle-aged women. It was not thought desirable in those days to have anyone as a nurse who had pretensions to attractiveness. It was felt desirable to have people of middle age, and in that way to be able to keep discipline and decency in those public institutions. When in 1869, having tired of the experience of the difficulties and troubles resulting from such service, I persuaded the committee of the Queen's Hospital to start a system of training nurses, and to take younger women to train, it was thought by some to be an unwise experiment which was not free from danger, seeing that the hospital was attached to a medical school. My answer was that the existing troubles were largely due to forgetfulness of the fact that if you want to have people about you that you can trust, you had better have some who have the maximum of things that they prize and want to take care of, and then you need have no fear about moral or other difficulties of that kind. The difficulty was to get started. I saw Mrs. Wardroper, of St. Thomas's Hospital, and we discussed the plan and how best to attract suitable candidates. On my suggestion we divided the county newspapers between us and advertised for farmers' and tradesmen's daughters to come and be trained as nurses. We were to take them for a year and compare notes at the end of the year. At the end of the year we had each had about 100 applications, with the result that Mrs. Wardroper succeeded at St. Thomas's Hospital in having four trained nurses out of her 100, and I had two at Birmingham. That result will show you the difficulties. At the end of 1877 I

wrote a letter to the *Times*, in which I advocated the training of nurses, and the nursing profession as a career for gentlewomen. I was answered in the *Times* by two eminent leaders of women, Miss Emily Shirreff and another lady, and the idea was that I was quite incorrigible; in fact, to talk of nursing as a career and profession for women was a "ghastly irony and a grotesque absurdity." It was hinted indeed that I belonged to the type of man who beats his wife. I am speaking of so recently as 1877, and I have the letters and articles among my most treasured possessions. However, we trained our nurses at Birmingham, gave them lectures and classes, and the result was that the Queen's Hospital became a place which was constantly visited. One day I was sitting in the office when a lady walked in; that lady was a famous personage in nursing; her life has been written, and she is well known. It was Sister Dora, a charming personality, who had her own opinions when she was at Walsall, where there is now a statue to her. She said, "I have come to see this place. Who are you?" I said, "I am the superintendent." "Oh!" she said. (I was then twenty-four years of age.) I said, "You are Sister Dora? I have not much to show you, but I will take you round the hospital." We went round the hospital. When we came back, she said to me, "It is not right." I said, "What is not right?" "These nurses you have here are too good-looking, and you are too young to be superintendent over them." It may interest you to hear of these little practical difficulties that we had at first, but in the end all our difficulties disappeared; there were no moral difficulties of any kind or description throughout the whole of that change and movement for getting the services of younger women, and we had as a result a perfect success in efficiency, in *morale*, in nursing, and in the provision for the patients. The New Nursing School was a thing which did so much good that it made the financial side of the hospital, which when we started was poor, extremely good.

Now take the position to-day. We have a multitude of nurses. In some hospitals there are less than three patients to a nurse, and increasing care is taken to train the nurses, educate them, and make them comfortable. The latest development is the Sister Tutor, who looks after the whole of the education of the nurses throughout their career. I have taken occasion to compare our work with that of the nurses in the United States, where the women are much better educated, if I may say so, than they are in this country, and the training is more scientific but not so practical. I find the Sister Tutor at St. Thomas's Hospital has attracted quite a high class of well-educated women to train as nurses. The thoroughness with which they do their work and the work they get out of the nurses are among the most important developments which the modern hospital has produced. You find improvements in nursing standards and *personnel* nearly everywhere as the Sister Tutor system grows. When we get the Royal College of British Nursing, which Mr. Arthur Stanley showed so much enterprise and courage in starting, and about which you will hear more soon, because they are going to raise a large fund for it, I think we shall find that the British nurse, who has held her own and does hold her own remarkably well, will soon be recognised as the first nurse in the world.

Look at the accommodation for nurses provided in 1868. One of the great difficulties we always had with the staff was the constant recurrent diphtheritic throat, which prevailed through the winter. It was a very troublesome and difficult thing indeed, but who can wonder at it? The accommodation at St. Bartholomew's Hospital for nurses, which is still very inadequate and poor, in 1868 gave some sisters for their room a cupboard which was suspended under the stairs. But in my hospital it was worse. Most of the nurses were put in the basement, in rooms fit for beer-cellars, for they had flagged floors with a central drain, and were washed down and mopped up. That condition of affairs required a great deal of fighting to get altered. Ultimately it was all changed and we obtained a new nurses' home. In addition to organising the training of the nurses and the employment of younger women, we had to secure better accommodation for them—but I am speaking to you in the Bedford Hospital; perhaps this had better not be said? (Laughter.) It is one of those things which I wrote about in my report on Bedford County Hospital. I have the chairman's authority for saying the present quarters for nurses will be removed, remedied, and changed for the better, immediately, *after the war*. Speaking of hospitals nowadays, so far as the general provision for nurses is concerned, it is frequently excellent; they have separate bedrooms and there is no putting three or five together in a room as there used to be. I am glad to see the disappearance of the cubicle. I have fought against its use for at least twenty-five years, and I hope I shall live to see the day when women who attend the sick will not have to occupy a cubicle. The larger and better nurses' homes are wonderful places, and the nurses are now able to live under conditions which should make them happy and reasonable people, but they are overworked. Everywhere the nurses are overworked in this country, and there is no one harder worked than the probationer. The strange thing is that the probationer is supposed to be a person who cannot learn in her first year unless she is kept constantly on her feet. There are Gorgons in many places, and there is in some hospitals what is known among nurses as the Gorgon-sister. If you take the sister's view, it is that probationers become young and flighty unless there is the wholesome dread of discipline.

So you have to keep, not a cheerful countenance, but a stern upper lip, and in that way you get the work done, and they learn their profession. I am speaking to women who are much better judges of that point than I am, but I think that too much stiffness in the upper lip in training our young men would mean, something very unpleasant to that upper lip.

